# 3,6-Di-*tert*-butyl-9-(quinolin-6-yl)-9*H*-carbazole

**DOI:** 10.1107/S1600536812030723

**Published:** 2012-08-01

**Authors:** Yun Chi, Lizhi Wang, Xiangxiang Li, Jianning Guan

**Affiliations:** a Department of Applied Chemistry, College of Science, Nanjing University of Technology, Nanjing 210009, People’s Republic of China

## Abstract

In the title compound, C_29_H_30_N_2_, the dihedral angle between the mean planes of the carbazole and the quinoline systems is 52.41 (6)°. Mol­ecules are linked into dimers by pairs of inter­molecular C—H⋯N hydrogen bonds and into a three-dimensional network by C—H⋯π inter­actions.

## Related literature

The title compound is an important inter­mediate in manufacturing materials such as organic light-emitting devices. For background to this class of compounds, see: Owczarczyk (2005 ▶). For the synthesis of the title compound, see: Muci & Buchwald (2002 ▶). For bond-length data, see: Allen *et al.* (1987 ▶).
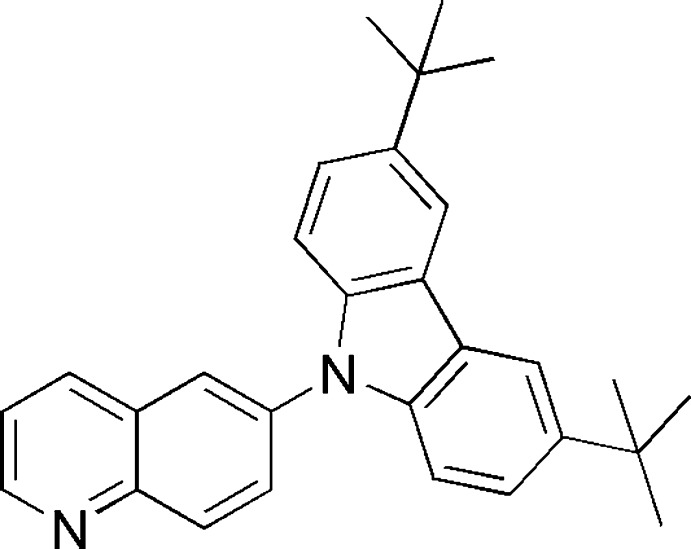


## Experimental

### 

#### Crystal data

C_29_H_30_N_2_
*M* *_r_* = 406.55
Triclinic, 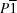

*a* = 5.9140 (12) Å
*b* = 13.133 (3) Å
*c* = 16.285 (3) Å
α = 69.30 (3)°
β = 83.28 (3)°
γ = 79.11 (3)°
*V* = 1160.1 (4) Å^3^
*Z* = 2
Mo *K*α radiation
μ = 0.07 mm^−1^
*T* = 293 K
0.30 × 0.20 × 0.10 mm

#### Data collection

Enraf–Nonius CAD-4 diffractometer
Absorption correction: ψ scan (North *et al.*, 1968 ▶) *T*_min_ = 0.980, *T*_max_ = 0.993
4710 measured reflections
4260 independent reflections
2838 reflections with *I* > 2σ(*I*)
*R*_int_ = 0.025
3 standard reflections every 200 reflections intensity decay: 1%

#### Refinement

*R*[*F* ^2^ > 2σ(*F* ^2^)] = 0.055
*wR*(*F* ^2^) = 0.171
*S* = 1.00
4260 reflections
281 parameters
H-atom parameters constrained
Δρ_max_ = 0.18 e Å^−3^
Δρ_min_ = −0.19 e Å^−3^

### 

Data collection: *CAD-4 EXPRESS* (Enraf–Nonius, 1994 ▶); cell refinement: *CAD-4 EXPRESS*; data reduction: *XCAD4* (Harms & Wocadlo, 1995 ▶); program(s) used to solve structure: *SHELXS97* (Sheldrick, 2008 ▶); program(s) used to refine structure: *SHELXL97* (Sheldrick, 2008 ▶); molecular graphics: *SHELXTL* (Sheldrick, 2008 ▶); software used to prepare material for publication: *PLATON* (Spek, 2009 ▶).

## Supplementary Material

Crystal structure: contains datablock(s) I, global. DOI: 10.1107/S1600536812030723/zq2170sup1.cif

Structure factors: contains datablock(s) I. DOI: 10.1107/S1600536812030723/zq2170Isup2.hkl

Supplementary material file. DOI: 10.1107/S1600536812030723/zq2170Isup3.cml

Additional supplementary materials:  crystallographic information; 3D view; checkCIF report

## Figures and Tables

**Table 1:** Hydrogen-bond geometry (Å, °) *Cg*1 is the centroid of the C1–C6 ring.

*D*—H⋯*A*	*D*—H	H⋯*A*	*D*⋯*A*	*D*—H⋯*A*
C3—H3*A*⋯N2^i^	0.93	2.70	3.625 (3)	172
C15—H15*B*⋯N2^i^	0.96	2.87	3.807 (4)	162
C29—H29*A*⋯*Cg*1^ii^	0.93	2.81	3.525 (3)	134

Symmetry codes: (i) 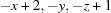; (ii) 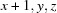.

**Fig. 1.**
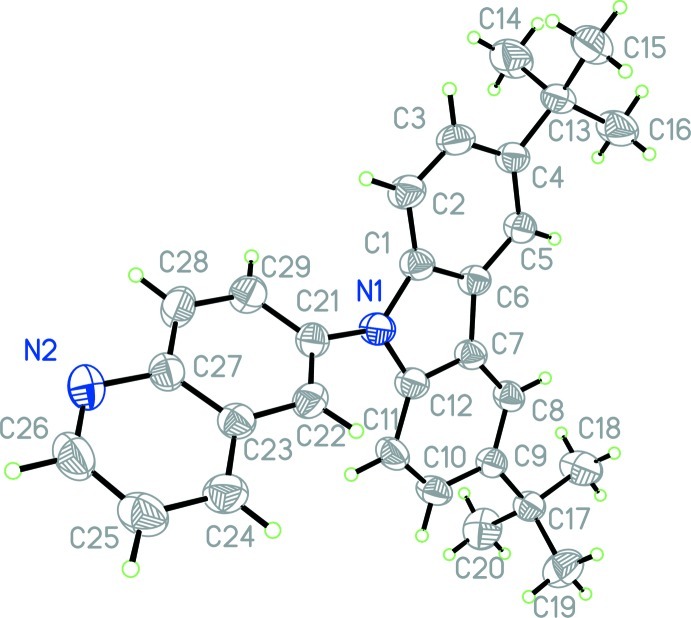
The molecular structure of the title compound with the atom-numbering scheme. Displacement ellipsoids are drawn at the 50% probability level.

**Fig. 2.**
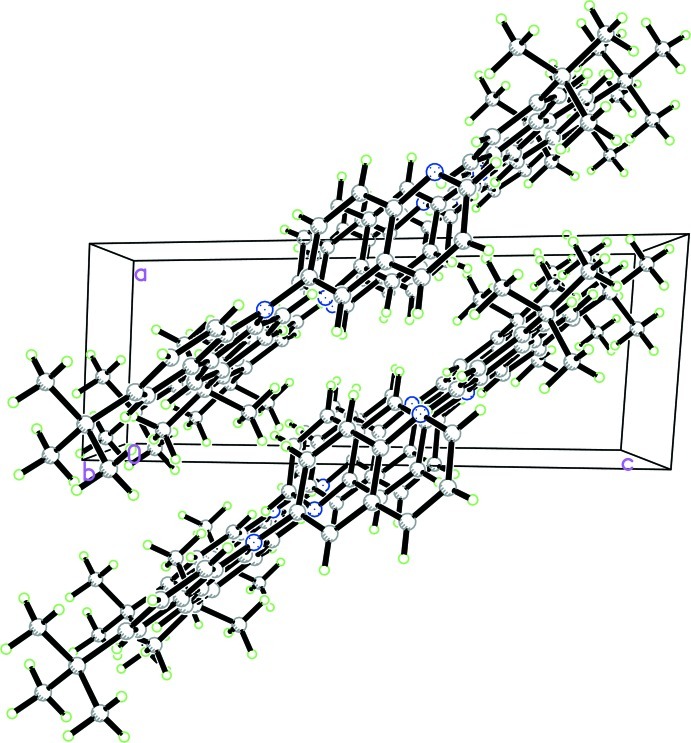
A packing diagram of the title compound viewed along the b axis.

## supplementary crystallographic information

### Comment

The title compound is an important intermediate for a kind of manufacturing material, such organic light-emitting devices (Owczarczyk, 2005) and particularly in the synthesis of (*Z*)-6-(3,6-di-*tert*-butyl-9*H*-carbazol-9-yl)-N-[6-(2,7-di-*tert*-butyl-9*H*-carbazol-9-yl)quinolin-2(1*H*)-ylidene]quinolin-2-amine.

In the title compound, C_29_H_30_N_2_, the dihedral angle between the mean planes of the carbazole and the quinoline rings is 52.41 (6)°. The bond lengths and angles are in normal ranges (Allen *et al.*, 1987). The molecules are linked into a dimer by pairs of intermolecular C—H···N hydrogen bonds (Table 1) and into a three-dimensional network by C–H···π interactions [ C29–H29A···Cg1*^ii^* = 2.81 Å, C25–H25A···Cg2*^iii^* = 3.19 Å; Cg1 and Cg2 are the centroids of the C1/C6 and C7/C12 rings, respectively; symmetry codes: *ii* = 1+x, y, z, *iii* = 1-x, 1-y, 1-z).

### Experimental

The title compound was prepared by a literature method (Muci & Buchwald, 2002). Crystals suitable for X-ray analysis were obtained by dissolving the title compound (0.41 g, 0.1 mmol) in acetonitrile (25 ml) and evaporating the solvent slowly at room temperature for about 10 d.

### Refinement

All H atoms were placed geometricallyand refined as riding with C—H = 0.93 Å and *U*_iso_(H) = 1.2*U*_eq_(C) for aromatic H atoms, and with C—H = 0.96 Å and *U*_iso_(H) = 1.5*U*_eq_(C) for methyl H atoms.

### Crystal data

**Table:**

C_29_H_30_N_2_	*Z* = 2
*M**_r_* = 406.55	*F*(000) = 436
Triclinic, *P*1	*D*_x_ = 1.164 Mg m^−^^3^
Hall symbol: -P 1	Mo *K*α radiation, λ = 0.71073 Å
*a* = 5.9140 (12) Å	Cell parameters from 25 reflections
*b* = 13.133 (3) Å	θ = 9–13°
*c* = 16.285 (3) Å	µ = 0.07 mm^−^^1^
α = 69.30 (3)°	*T* = 293 K
β = 83.28 (3)°	Acicular, colourless
γ = 79.11 (3)°	0.30 × 0.20 × 0.10 mm
*V* = 1160.1 (4) Å^3^	

### Data collection

**Table:**

Enraf–Nonius CAD-4 diffractometer	2838 reflections with *I* > 2σ(*I*)
Radiation source: fine-focus sealed tube	*R*_int_ = 0.025
Graphite monochromator	θ_max_ = 25.4°, θ_min_ = 1.3°
ω/2θ scans	*h* = 0→7
Absorption correction: ψ scan (North *et al.*, 1968)	*k* = −15→15
*T*_min_ = 0.980, *T*_max_ = 0.993	*l* = −19→19
4710 measured reflections	3 standard reflections every 200 reflections
4260 independent reflections	intensity decay: 1%

### Refinement

**Table:**

Refinement on *F*^2^	Secondary atom site location: difference Fourier map
Least-squares matrix: full	Hydrogen site location: inferred from neighbouring sites
*R*[*F*^2^ > 2σ(*F*^2^)] = 0.055	H-atom parameters constrained
*wR*(*F*^2^) = 0.171	*w* = 1/[σ^2^(*F*_o_^2^) + (0.1*P*)^2^] where *P* = (*F*_o_^2^ + 2*F*_c_^2^)/3
*S* = 1.00	(Δ/σ)_max_ < 0.001
4260 reflections	Δρ_max_ = 0.18 e Å^−^^3^
281 parameters	Δρ_min_ = −0.19 e Å^−^^3^
0 restraints	Extinction correction: *SHELXL97* (Sheldrick, 2008), Fc^*^=kFc[1+0.001xFc^2^λ^3^/sin(2θ)]^-1/4^
Primary atom site location: structure-invariant direct methods	Extinction coefficient: 0.094 (8)

### Special details

**Table:**

Geometry. All e.s.d.'s (except the e.s.d. in the dihedral angle between two l.s. planes) are estimated using the full covariance matrix. The cell e.s.d.'s are taken into account individually in the estimation of e.s.d.'s in distances, angles and torsion angles; correlations between e.s.d.'s in cell parameters are only used when they are defined by crystal symmetry. An approximate (isotropic) treatment of cell e.s.d.'s is used for estimating e.s.d.'s involving l.s. planes.
Refinement. Refinement of *F*^2^ against ALL reflections. The weighted *R*-factor *wR* and goodness of fit *S* are based on *F*^2^, conventional *R*-factors *R* are based on *F*, with *F* set to zero for negative *F*^2^. The threshold expression of *F*^2^ > σ(*F*^2^) is used only for calculating *R*-factors(gt) *etc*. and is not relevant to the choice of reflections for refinement. *R*-factors based on *F*^2^ are statistically about twice as large as those based on *F*, and *R*- factors based on ALL data will be even larger.

### Fractional atomic coordinates and isotropic or equivalent isotropic displacement parameters (Å^2^)

**Table:**

	*x*	*y*	*z*	*U*_iso_*/*U*_eq_	
N1	0.6826 (3)	0.28889 (14)	0.32604 (12)	0.0430 (5)	
C1	0.6135 (4)	0.20305 (17)	0.30806 (14)	0.0386 (5)	
N2	1.2433 (3)	0.27066 (16)	0.58530 (13)	0.0488 (5)	
C2	0.6628 (4)	0.08984 (18)	0.34991 (15)	0.0448 (6)	
H2B	0.7574	0.0598	0.3972	0.054*	
C3	0.5675 (4)	0.02372 (18)	0.31929 (15)	0.0456 (6)	
H3A	0.6011	−0.0523	0.3466	0.055*	
C4	0.4209 (4)	0.06508 (17)	0.24845 (14)	0.0394 (5)	
C5	0.3708 (4)	0.17821 (17)	0.20890 (14)	0.0378 (5)	
H5A	0.2732	0.2081	0.1625	0.045*	
C6	0.4661 (4)	0.24780 (16)	0.23841 (13)	0.0359 (5)	
C7	0.4423 (4)	0.36640 (16)	0.21274 (14)	0.0371 (5)	
C8	0.3217 (4)	0.45393 (17)	0.14801 (14)	0.0386 (5)	
H8A	0.2291	0.4396	0.1123	0.046*	
C9	0.3386 (4)	0.56156 (17)	0.13664 (14)	0.0379 (5)	
C10	0.4805 (4)	0.57927 (18)	0.19202 (15)	0.0448 (6)	
H10A	0.4941	0.6515	0.1844	0.054*	
C11	0.6006 (4)	0.49529 (18)	0.25698 (15)	0.0456 (6)	
H11A	0.6935	0.5100	0.2924	0.055*	
C12	0.5782 (4)	0.38803 (17)	0.26771 (14)	0.0404 (6)	
C13	0.3314 (4)	−0.01504 (18)	0.21547 (16)	0.0458 (6)	
C14	0.5381 (5)	−0.0732 (2)	0.1730 (2)	0.0707 (9)	
H14A	0.6057	−0.0193	0.1245	0.106*	
H14B	0.4863	−0.1243	0.1522	0.106*	
H14C	0.6511	−0.1122	0.2158	0.106*	
C15	0.2244 (5)	−0.1018 (2)	0.29260 (19)	0.0657 (8)	
H15A	0.0963	−0.0660	0.3196	0.099*	
H15B	0.3380	−0.1413	0.3350	0.099*	
H15C	0.1719	−0.1524	0.2714	0.099*	
C16	0.1486 (5)	0.0432 (2)	0.1483 (2)	0.0692 (9)	
H16A	0.2121	0.0976	0.0990	0.104*	
H16B	0.0186	0.0784	0.1749	0.104*	
H16C	0.1000	−0.0098	0.1287	0.104*	
C17	0.2100 (4)	0.66163 (17)	0.06702 (14)	0.0420 (6)	
C18	0.0785 (5)	0.6271 (2)	0.00882 (18)	0.0705 (9)	
H18A	0.1842	0.5838	−0.0201	0.106*	
H18B	0.0028	0.6915	−0.0344	0.106*	
H18C	−0.0346	0.5840	0.0443	0.106*	
C19	0.0372 (5)	0.7300 (2)	0.11289 (18)	0.0647 (8)	
H19A	−0.0719	0.6854	0.1496	0.097*	
H19B	−0.0428	0.7927	0.0695	0.097*	
H19C	0.1182	0.7546	0.1483	0.097*	
C20	0.3835 (5)	0.7328 (2)	0.00850 (17)	0.0672 (8)	
H20A	0.4914	0.6903	−0.0204	0.101*	
H20B	0.4649	0.7573	0.0439	0.101*	
H20C	0.3033	0.7957	−0.0348	0.101*	
C21	0.8180 (4)	0.27973 (17)	0.39558 (14)	0.0379 (5)	
C22	0.7372 (4)	0.33765 (17)	0.45071 (14)	0.0405 (6)	
H22A	0.5888	0.3776	0.4455	0.049*	
C23	0.8755 (4)	0.33782 (16)	0.51539 (14)	0.0368 (5)	
C24	0.8046 (4)	0.39882 (19)	0.57257 (15)	0.0478 (6)	
H24A	0.6586	0.4410	0.5694	0.057*	
C25	0.9498 (5)	0.3956 (2)	0.63199 (16)	0.0523 (7)	
H25A	0.9061	0.4359	0.6697	0.063*	
C26	1.1674 (5)	0.3302 (2)	0.63553 (16)	0.0523 (7)	
H26A	1.2655	0.3290	0.6766	0.063*	
C27	1.0984 (4)	0.27437 (17)	0.52467 (14)	0.0383 (5)	
C28	1.1730 (4)	0.2113 (2)	0.46923 (15)	0.0471 (6)	
H28A	1.3166	0.1667	0.4763	0.056*	
C29	1.0385 (4)	0.21466 (19)	0.40572 (15)	0.0452 (6)	
H29A	1.0922	0.1739	0.3689	0.054*	

### Atomic displacement parameters (Å^2^)

**Table:**

	*U*^11^	*U*^22^	*U*^33^	*U*^12^	*U*^13^	*U*^23^
N1	0.0558 (12)	0.0340 (10)	0.0408 (11)	−0.0017 (9)	−0.0206 (9)	−0.0117 (8)
C1	0.0461 (14)	0.0355 (12)	0.0361 (12)	−0.0047 (10)	−0.0069 (10)	−0.0138 (10)
N2	0.0479 (12)	0.0545 (12)	0.0479 (12)	−0.0061 (10)	−0.0150 (10)	−0.0194 (10)
C2	0.0548 (15)	0.0363 (12)	0.0413 (13)	0.0005 (11)	−0.0176 (11)	−0.0101 (10)
C3	0.0544 (15)	0.0311 (12)	0.0492 (14)	−0.0012 (10)	−0.0117 (12)	−0.0111 (10)
C4	0.0435 (13)	0.0344 (12)	0.0415 (12)	−0.0049 (10)	−0.0041 (10)	−0.0146 (10)
C5	0.0449 (13)	0.0355 (12)	0.0336 (12)	−0.0074 (10)	−0.0081 (10)	−0.0103 (10)
C6	0.0420 (13)	0.0325 (11)	0.0321 (11)	−0.0040 (9)	−0.0060 (10)	−0.0094 (9)
C7	0.0437 (13)	0.0335 (12)	0.0358 (12)	−0.0052 (10)	−0.0083 (10)	−0.0124 (10)
C8	0.0449 (13)	0.0379 (12)	0.0357 (12)	−0.0079 (10)	−0.0107 (10)	−0.0126 (10)
C9	0.0430 (13)	0.0346 (12)	0.0364 (12)	−0.0066 (10)	−0.0069 (10)	−0.0108 (9)
C10	0.0583 (15)	0.0306 (11)	0.0470 (14)	−0.0095 (11)	−0.0118 (12)	−0.0111 (10)
C11	0.0550 (15)	0.0392 (13)	0.0485 (14)	−0.0099 (11)	−0.0203 (12)	−0.0155 (11)
C12	0.0485 (14)	0.0339 (12)	0.0395 (12)	−0.0038 (10)	−0.0118 (11)	−0.0119 (10)
C13	0.0513 (15)	0.0328 (12)	0.0564 (15)	−0.0094 (10)	−0.0099 (12)	−0.0152 (11)
C14	0.078 (2)	0.0668 (18)	0.087 (2)	−0.0156 (16)	0.0004 (17)	−0.0499 (17)
C15	0.0677 (19)	0.0491 (15)	0.0773 (19)	−0.0214 (14)	−0.0127 (15)	−0.0088 (14)
C16	0.086 (2)	0.0496 (15)	0.079 (2)	−0.0136 (15)	−0.0397 (17)	−0.0181 (14)
C17	0.0491 (14)	0.0353 (12)	0.0403 (13)	−0.0048 (10)	−0.0117 (11)	−0.0093 (10)
C18	0.099 (2)	0.0450 (15)	0.0674 (18)	−0.0024 (15)	−0.0489 (18)	−0.0091 (13)
C19	0.0633 (18)	0.0584 (17)	0.0654 (18)	0.0094 (14)	−0.0124 (15)	−0.0196 (14)
C20	0.0714 (19)	0.0586 (17)	0.0534 (16)	−0.0104 (14)	−0.0081 (14)	0.0048 (13)
C21	0.0425 (13)	0.0364 (12)	0.0355 (12)	−0.0047 (10)	−0.0106 (10)	−0.0111 (10)
C22	0.0407 (13)	0.0366 (12)	0.0421 (13)	0.0019 (10)	−0.0115 (10)	−0.0121 (10)
C23	0.0440 (13)	0.0315 (11)	0.0335 (11)	−0.0066 (10)	−0.0056 (10)	−0.0077 (9)
C24	0.0556 (15)	0.0439 (13)	0.0465 (14)	0.0009 (11)	−0.0088 (12)	−0.0212 (11)
C25	0.0711 (18)	0.0500 (14)	0.0427 (14)	−0.0105 (13)	−0.0072 (13)	−0.0224 (12)
C26	0.0613 (17)	0.0558 (15)	0.0473 (14)	−0.0158 (13)	−0.0147 (13)	−0.0198 (12)
C27	0.0388 (13)	0.0387 (12)	0.0378 (12)	−0.0084 (10)	−0.0053 (10)	−0.0111 (10)
C28	0.0387 (13)	0.0548 (15)	0.0492 (14)	0.0040 (11)	−0.0104 (11)	−0.0229 (12)
C29	0.0465 (14)	0.0512 (14)	0.0423 (13)	0.0004 (11)	−0.0043 (11)	−0.0248 (11)

### Geometric parameters (Å, º)

**Table:**

N1—C12	1.392 (3)	C15—H15B	0.9600
N1—C1	1.400 (3)	C15—H15C	0.9600
N1—C21	1.420 (3)	C16—H16A	0.9600
C1—C2	1.388 (3)	C16—H16B	0.9600
C1—C6	1.399 (3)	C16—H16C	0.9600
N2—C26	1.310 (3)	C17—C18	1.523 (3)
N2—C27	1.364 (3)	C17—C20	1.530 (3)
C2—C3	1.370 (3)	C17—C19	1.535 (3)
C2—H2B	0.9300	C18—H18A	0.9600
C3—C4	1.411 (3)	C18—H18B	0.9600
C3—H3A	0.9300	C18—H18C	0.9600
C4—C5	1.383 (3)	C19—H19A	0.9600
C4—C13	1.534 (3)	C19—H19B	0.9600
C5—C6	1.397 (3)	C19—H19C	0.9600
C5—H5A	0.9300	C20—H20A	0.9600
C6—C7	1.446 (3)	C20—H20B	0.9600
C7—C8	1.397 (3)	C20—H20C	0.9600
C7—C12	1.401 (3)	C21—C22	1.363 (3)
C8—C9	1.381 (3)	C21—C29	1.410 (3)
C8—H8A	0.9300	C22—C23	1.408 (3)
C9—C10	1.404 (3)	C22—H22A	0.9300
C9—C17	1.537 (3)	C23—C24	1.412 (3)
C10—C11	1.377 (3)	C23—C27	1.413 (3)
C10—H10A	0.9300	C24—C25	1.352 (3)
C11—C12	1.387 (3)	C24—H24A	0.9300
C11—H11A	0.9300	C25—C26	1.401 (4)
C13—C16	1.524 (3)	C25—H25A	0.9300
C13—C15	1.533 (3)	C26—H26A	0.9300
C13—C14	1.543 (4)	C27—C28	1.412 (3)
C14—H14A	0.9600	C28—C29	1.361 (3)
C14—H14B	0.9600	C28—H28A	0.9300
C14—H14C	0.9600	C29—H29A	0.9300
C15—H15A	0.9600		
			
C12—N1—C1	107.81 (17)	C13—C16—H16A	109.5
C12—N1—C21	124.67 (18)	C13—C16—H16B	109.5
C1—N1—C21	127.31 (18)	H16A—C16—H16B	109.5
C2—C1—C6	120.8 (2)	C13—C16—H16C	109.5
C2—C1—N1	130.0 (2)	H16A—C16—H16C	109.5
C6—C1—N1	109.11 (18)	H16B—C16—H16C	109.5
C26—N2—C27	116.8 (2)	C18—C17—C20	108.3 (2)
C3—C2—C1	117.8 (2)	C18—C17—C19	108.5 (2)
C3—C2—H2B	121.1	C20—C17—C19	109.4 (2)
C1—C2—H2B	121.1	C18—C17—C9	111.84 (18)
C2—C3—C4	123.3 (2)	C20—C17—C9	109.40 (19)
C2—C3—H3A	118.3	C19—C17—C9	109.40 (19)
C4—C3—H3A	118.3	C17—C18—H18A	109.5
C5—C4—C3	117.8 (2)	C17—C18—H18B	109.5
C5—C4—C13	122.3 (2)	H18A—C18—H18B	109.5
C3—C4—C13	119.84 (19)	C17—C18—H18C	109.5
C4—C5—C6	120.2 (2)	H18A—C18—H18C	109.5
C4—C5—H5A	119.9	H18B—C18—H18C	109.5
C6—C5—H5A	119.9	C17—C19—H19A	109.5
C5—C6—C1	120.00 (19)	C17—C19—H19B	109.5
C5—C6—C7	133.03 (19)	H19A—C19—H19B	109.5
C1—C6—C7	106.96 (18)	C17—C19—H19C	109.5
C8—C7—C12	119.73 (19)	H19A—C19—H19C	109.5
C8—C7—C6	133.6 (2)	H19B—C19—H19C	109.5
C12—C7—C6	106.66 (18)	C17—C20—H20A	109.5
C9—C8—C7	120.6 (2)	C17—C20—H20B	109.5
C9—C8—H8A	119.7	H20A—C20—H20B	109.5
C7—C8—H8A	119.7	C17—C20—H20C	109.5
C8—C9—C10	117.7 (2)	H20A—C20—H20C	109.5
C8—C9—C17	123.27 (19)	H20B—C20—H20C	109.5
C10—C9—C17	119.00 (19)	C22—C21—C29	119.9 (2)
C11—C10—C9	123.4 (2)	C22—C21—N1	119.58 (19)
C11—C10—H10A	118.3	C29—C21—N1	120.43 (19)
C9—C10—H10A	118.3	C21—C22—C23	120.9 (2)
C10—C11—C12	117.7 (2)	C21—C22—H22A	119.6
C10—C11—H11A	121.2	C23—C22—H22A	119.6
C12—C11—H11A	121.2	C22—C23—C24	123.6 (2)
C11—C12—N1	129.6 (2)	C22—C23—C27	119.3 (2)
C11—C12—C7	120.9 (2)	C24—C23—C27	117.1 (2)
N1—C12—C7	109.45 (18)	C25—C24—C23	119.7 (2)
C16—C13—C15	107.9 (2)	C25—C24—H24A	120.1
C16—C13—C4	112.41 (19)	C23—C24—H24A	120.1
C15—C13—C4	110.1 (2)	C24—C25—C26	118.6 (2)
C16—C13—C14	109.3 (2)	C24—C25—H25A	120.7
C15—C13—C14	109.1 (2)	C26—C25—H25A	120.7
C4—C13—C14	107.97 (19)	N2—C26—C25	124.9 (2)
C13—C14—H14A	109.5	N2—C26—H26A	117.5
C13—C14—H14B	109.5	C25—C26—H26A	117.5
H14A—C14—H14B	109.5	N2—C27—C28	118.7 (2)
C13—C14—H14C	109.5	N2—C27—C23	122.9 (2)
H14A—C14—H14C	109.5	C28—C27—C23	118.4 (2)
H14B—C14—H14C	109.5	C29—C28—C27	121.1 (2)
C13—C15—H15A	109.5	C29—C28—H28A	119.4
C13—C15—H15B	109.5	C27—C28—H28A	119.4
H15A—C15—H15B	109.5	C28—C29—C21	120.2 (2)
C13—C15—H15C	109.5	C28—C29—H29A	119.9
H15A—C15—H15C	109.5	C21—C29—H29A	119.9
H15B—C15—H15C	109.5		
			
C12—N1—C1—C2	−177.2 (2)	C6—C7—C12—N1	−0.4 (3)
C21—N1—C1—C2	−2.3 (4)	C5—C4—C13—C16	−11.1 (3)
C12—N1—C1—C6	0.1 (3)	C3—C4—C13—C16	171.5 (2)
C21—N1—C1—C6	175.0 (2)	C5—C4—C13—C15	−131.5 (2)
C6—C1—C2—C3	1.7 (3)	C3—C4—C13—C15	51.1 (3)
N1—C1—C2—C3	178.7 (2)	C5—C4—C13—C14	109.5 (3)
C1—C2—C3—C4	−0.7 (4)	C3—C4—C13—C14	−67.9 (3)
C2—C3—C4—C5	−0.5 (4)	C8—C9—C17—C18	−4.2 (3)
C2—C3—C4—C13	177.0 (2)	C10—C9—C17—C18	175.9 (2)
C3—C4—C5—C6	0.8 (3)	C8—C9—C17—C20	−124.2 (2)
C13—C4—C5—C6	−176.7 (2)	C10—C9—C17—C20	55.9 (3)
C4—C5—C6—C1	0.2 (3)	C8—C9—C17—C19	116.0 (2)
C4—C5—C6—C7	−178.1 (2)	C10—C9—C17—C19	−63.9 (3)
C2—C1—C6—C5	−1.5 (3)	C12—N1—C21—C22	48.2 (3)
N1—C1—C6—C5	−179.06 (19)	C1—N1—C21—C22	−125.9 (2)
C2—C1—C6—C7	177.2 (2)	C12—N1—C21—C29	−129.4 (2)
N1—C1—C6—C7	−0.4 (2)	C1—N1—C21—C29	56.5 (3)
C5—C6—C7—C8	−2.2 (4)	C29—C21—C22—C23	3.1 (3)
C1—C6—C7—C8	179.4 (2)	N1—C21—C22—C23	−174.45 (19)
C5—C6—C7—C12	178.9 (2)	C21—C22—C23—C24	177.9 (2)
C1—C6—C7—C12	0.5 (2)	C21—C22—C23—C27	−1.9 (3)
C12—C7—C8—C9	1.2 (3)	C22—C23—C24—C25	−179.0 (2)
C6—C7—C8—C9	−177.6 (2)	C27—C23—C24—C25	0.8 (3)
C7—C8—C9—C10	0.1 (3)	C23—C24—C25—C26	−0.6 (4)
C7—C8—C9—C17	−179.8 (2)	C27—N2—C26—C25	0.8 (4)
C8—C9—C10—C11	−0.6 (4)	C24—C25—C26—N2	−0.2 (4)
C17—C9—C10—C11	179.2 (2)	C26—N2—C27—C28	−179.9 (2)
C9—C10—C11—C12	−0.2 (4)	C26—N2—C27—C23	−0.6 (3)
C10—C11—C12—N1	178.4 (2)	C22—C23—C27—N2	179.6 (2)
C10—C11—C12—C7	1.5 (4)	C24—C23—C27—N2	−0.2 (3)
C1—N1—C12—C11	−177.0 (2)	C22—C23—C27—C28	−1.0 (3)
C21—N1—C12—C11	8.0 (4)	C24—C23—C27—C28	179.1 (2)
C1—N1—C12—C7	0.2 (3)	N2—C27—C28—C29	−177.9 (2)
C21—N1—C12—C7	−174.9 (2)	C23—C27—C28—C29	2.8 (3)
C8—C7—C12—C11	−2.1 (3)	C27—C28—C29—C21	−1.6 (4)
C6—C7—C12—C11	177.0 (2)	C22—C21—C29—C28	−1.4 (4)
C8—C7—C12—N1	−179.5 (2)	N1—C21—C29—C28	176.1 (2)

### Hydrogen-bond geometry (Å, º)

Cg1 is the centroid of the C1–C6 ring.

**Table:**

*D*—H···*A*	*D*—H	H···*A*	*D*···*A*	*D*—H···*A*
C3—H3*A*···N2^i^	0.93	2.70	3.625 (3)	172
C15—H15*B*···N2^i^	0.96	2.87	3.807 (4)	162
C29—H29*A*···*Cg*1^ii^	0.93	2.81	3.525 (3)	134

Symmetry codes: (i) −*x*+2, −*y*, −*z*+1; (ii) *x*+1, *y*, *z*.
